# EEG-microstate dependent emergence of perceptual awareness

**DOI:** 10.3389/fnbeh.2014.00163

**Published:** 2014-05-14

**Authors:** Juliane Britz, Laura Díaz Hernàndez, Tony Ro, Christoph M. Michel

**Affiliations:** ^1^Department of Fundamental Neuroscience, Centre Médical Universitaire, University of GenevaGeneva, Switzerland; ^2^EEG Brain Mapping Core, Center for Biomedical Imaging (CIBM), University of GenevaGeneva, Switzerland; ^3^Department of Psychiatric Neurophysiology, University Hospital of PsychiatryBern, Switzerland; ^4^Department of Psychology, The City College and Graduate Center, City University of New YorkNew York, NY, USA

**Keywords:** EEG microstates, perceptual awareness, meta-contrast masking, LAURA inverse solution, alpha phase

## Abstract

We investigated whether the differences in perceptual awareness for stimuli at the threshold of awareness can arise from different global brain states before stimulus onset indexed by the EEG microstate. We used a metacontrast backward masking paradigm in which subjects had to discriminate between two weak stimuli and obtained measures of accuracy and awareness while their EEG was recorded from 256 channels. Comparing targets that were correctly identified with and without awareness allowed us to contrast differences in awareness while keeping performance constant for identical physical stimuli. Two distinct pre-stimulus scalp potential fields (microstate maps) dissociated correct identification with and without awareness, and their estimated intracranial generators were stronger in primary visual cortex before correct identification without awareness. This difference in activity cannot be explained by differences in alpha power or phase which were less reliably linked with differential pre-stimulus activation of primary visual cortex. Our results shed a new light on the function of pre-stimulus activity in early visual cortex in visual awareness and emphasize the importance of trial-by-trials analysis of the spatial configuration of the scalp potential field identified with multichannel EEG.

## Introduction

Under certain circumstances, sensation and perception can be dissociated such that the same physical stimulus gives rise to different perceptual outcomes. Phenomena like multi-stable perception (e.g., the Necker cube and binocularly rivalry) or stimuli presented at perceptual thresholds share the fact that the same stimulus can be perceived one way or another or that it can either be perceived or not. These conditions allow us to study perceptual awareness independent of sensory processing. Since such differences in perceptual awareness cannot arise from physical differences in the stimuli, they might arise from differences in the brain state before the stimulus is encountered (for a recent review see Britz and Michel, 2011).

Imaging techniques with high temporal resolution, such as EEG and MEG, provide a means of distinguishing pre-stimulus activity from post-stimulus activity. The EEG measures the electrical field generated by the brain by using electrodes placed across the scalp to differentially measure the summation of all concurrently active intracranial sources at a given time point. The EEG measurement can be considered as a matrix with space in one dimension and time in the other dimension. The analyses of the EEG can focus on the temporal dimension and assess differences in frequency power or phase at selected electrodes, or it can focus on the spatial dimension and assess topographic differences of the electric field. Both characteristics of the EEG have been shown to vary before stimulus onset and to influence how upcoming stimuli can be treated and perceived.

Differences in perceptual awareness of stimuli presented at the detection and discrimination thresholds could be related to differences in pre-stimulus power and phase in the alpha frequency band. The alpha band comprises frequencies between 8 and 12 Hz, and its functional significance has been most commonly described by reflecting cortical excitability to which its power is inversely related (Pfurtscheller, 1992), with higher levels of alpha power corresponding to lower levels of excitability, and vice versa. In line with this notion, it has been shown that the detection of a light pulse presented at the sensory threshold depends on the pre-stimulus alpha power: undetected stimuli were preceded by increased alpha power compared to detected stimuli (Ergenoglu et al., 2004). Likewise, illusory visual percepts (phosphenes) induced by a TMS-pulse have been shown to depend on both inter- (Romei et al., 2008b) and intra-individual (Romei et al., 2008a) differences in pre-stimulus alpha power. In addition to detection, discrimination ability in a backward masking task has been related to both inter- (Hanslmayr et al., 2005) and intra-(Hanslmayr et al., 2007; van Dijk et al., 2008) individual differences in pre-stimulus alpha power. Also, the perceptual reversals of a Necker cube have been shown to be preceded by decreased alpha power (Isoğlu-Alkaç et al., 1998, 2000; Isoğlu-Alkaç and Strüber, 2006). Similarly, perceptual reversals during binocular rivalry are preceded by decreased gamma power (Doesburg et al., 2005). In addition, the detection of a near-threshold stimulus (Busch et al., 2009) and the efficiency of metacontrast masking (Mathewson et al., 2009) have also been shown to be related to local differences in the pre-stimulus alpha phase. Taken together, these results suggest that the ability to detect and discriminate stimuli presented at the perceptual threshold can vary as a function of the pre-stimulus alpha power and phase, and, hence, on the excitability of early visual cortex through pulsed inhibition (Mathewson et al., 2011).

The problem is that amplitude, power and phase modulations of EEG waveforms are *local* measures that vary with the reference; in addition, amplitude and power modulations vary at every instant, and the phase is different at every electrode (Lehmann and Michel, 1989), which makes it difficult to interpret the physiological meaning of local differences in power or phase between conditions. The EEG scalp potential field on the other hand is a *global* and *reference-free* measure of overall brain activity. Different topographies of the potential field directly indicate differences in the configuration of the underlying sources (Helmholtz, 1853; Vaughan, 1982). Unlike the constantly changing amplitude and power modulations, the configuration of the scalp topography remains stable for brief periods (∼80–120 ms) with sharp transitions between subsequent states. These brief states of stable topography have been named the “EEG microstates”. Microstates have been shown to characterize the contents of spontaneous thoughts (Lehmann et al., 1998, 2010), to explain the trial-to-trial differences in the hemispheric lateralization of emotional word processing (Mohr et al., 2005) and to determine the topography of ERPs (Kondákor et al., 1995; Kondakor et al., 1997). We have recently shown that microstates can be considered the electrophysiological correlate of resting-state networks identified with fMRI which suggests that the momentary scalp configuration represents the activity in a specific neurocognitive network (Britz et al., 2010; Van de Ville et al., 2010).

More recently, we have started to investigate the notion that the global state of the brain indexed by the pre-stimulus microstate can determine the perceptual awareness of multi-stable stimuli. These stimuli are physically identical but can have different perceptual interpretations. We could show that the perceptual reversals of ambiguous figures (Britz et al., 2009) and during binocular rivalry (Britz et al., 2011) arise as a direct consequence of the pre-stimulus microstate. In both studies, stimuli were presented intermittently, and we identified two microstate topographies immediately before stimulus onset that dissociated perceptual reversals from perceptual stability. Statistical parametric mapping of their concomitant source differences showed that the reversals were caused by increased neuronal activity in the right inferior parietal lobe in both cases.

Microstates have not yet been used to investigate the emergence of perceptual awareness at sensory thresholds. Meta-contrast masking is a powerful technique for experimentally manipulating the visibility of stimuli. A briefly presented target stimulus is followed by a mask with the same inner cutout of the same contour as the stimulus. The visibility of the target varies as a U-shaped function of the interval between stimulus and mask: at very brief and long inter-stimulus intervals (ISIs), the target is visible, but at intermediate ISIs, the mask efficiently renders the target invisible. Moreover, within those intermediate ISIs, there appears to be a “sweet spot” at which the masking effect is efficient in roughly 50% of cases, i.e., the same stimulus is perceived in about half the trials and not perceived in the other half of the trials. The efficiency of making is commonly explained by disruption of re-entrant processing between higher and lower visual areas after stimulus onset (Fahrenfort et al., 2007, 2008) and recurrent processing within early visual areas (Boehler et al., 2008). We assessed a different hypothesis, namely that perceptual awareness and the efficiency of masking might depend on the global brain state at the time of stimulus arrival.

In the present study, we used Electrical Neuroimaging (Murray et al., 2008; Michel et al., 2009) to investigate whether differences in perceptual awareness can arise from differences in the pre-stimulus brain state indexed by the EEG microstate immediately before stimulus onset. We used a metacontrast backward masking paradigm where subjects had to discriminate between two targets and assessed differences in subjective awareness while performance was kept constant for physically identical stimuli. We compared the same physical stimulus when it was correctly identified with and without awareness, and equating performance and stimulus properties avoided the confound of awareness with performance and stimulus properties. Subjective awareness and objective performance have been shown to be independent, and awareness is not necessary for correct performance (Schwiedrzik et al., 2011).

We hypothesized that different pre-stimulus microstates and thus different neuronal networks in the brain are active when subjects will become aware of a stimulus in a given trial than when they do not, and our goal was to identify two states that dissociate correct stimulus identification with and without awareness. Statistical parametric mapping of their concomitant intracranial generator differences will then reveal the location of activity differences for stimuli that were correctly identified with and without awareness. In addition to the global measure of pre-stimulus microstates, we investigated local differences in alpha power and phase in order to relate our findings to those from previous studies. Pre-stimulus differences in alpha power have been independently related to performance and awareness. If pre-stimulus alpha power over visual cortex is related to visual awareness, we expect to find higher alpha power before stimuli that were correctly detected with than without awareness. Similarly, differences in alpha phase over occipital electrodes at stimulus onset should vary as a function of awareness.

## Materials and Methods

### Subjects

Twenty-three healthy adults (7 male, mean age 23,3 years, range 18–37) were initially screened for the EEG study, and a separate group of eight adults (3 male, mean age 28.87 years, range 22–37 years) participated in a behavioral pre-test. All subjects were right handed as assessed by the Edinburgh Handedness Inventory (Oldfield, 1971), had normal or corrected-to-normal visual acuity as assessed with the Freiburg Visual Acuity Test (Bach, 1996), the mean decimal Visual Acuity across subjects was 1.698. None of the subjects reported a history of psychiatric or neurological impairments. Subjects participated for monetary compensation of CHF 20/h after giving informed consent approved by the Ethics Committee of the University Hospital of Geneva. Eight participants did not participate in the EEG study because of their behavioral results in a training period (either too many aware or too many unaware trials). A total of 15 subjects (4 male, mean age: 23.81 years, range 19–37) completed the EEG experiment. The data from four subjects was excluded from the analysis because of primarily unaware responses in one case, primarily correct aware (CA) responses in another case, and chance performance and an insufficient number of acceptable trials due to data quality in the two other cases. Thus, the behavioral and EEG data from a total of 11 subjects were submitted to further analysis.

### Stimuli and Procedure

**Figure 1:**
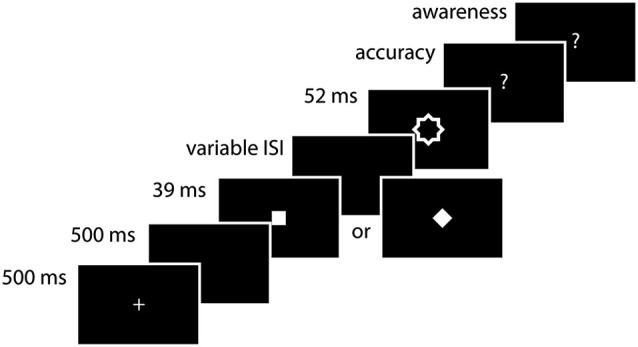
**Experimental procedure**. A diamond or square was presented for 39 ms. After a variable ISI (39, 52, 65, 104 ms), it was followed by a contour mask. Subjects first had to indicate which stimulus they saw (accuracy measurement) and then whether they saw the stimulus or whether they were guessing (awareness measurement).

Figure 1 illustrates the stimuli and experimental procedure. Target stimuli were a square and a diamond (the square rotated by 45°) subtending 1° of visual angle. The mask was a larger contour of the two superimposed targets, which subtended 2° of visual angle, with an inner cutout of the same contour. All stimuli were presented in white (67.21 cd/m^2^) on a black background in the center of a CRT screen with a refresh rate of 75 Hz. Stimulus presentation and timing was achieved using E-prime2 (Psychology Software Tools, Inc., Pittsburgh, USA).

Each trial began with the presentation of a fixation cross (1°) at the center of the screen for 500 ms. After a blank interval of 500 ms one of the two possible targets (square or diamond) was presented for 39 ms. The target was followed by a blank interval of variable duration (39, 52, 65, 104 ms). Subsequently, the mask was presented for 52 ms. After the offset of the mask, subjects first had to indicate which target stimulus they saw, yielding a measurement of accuracy. They then had to indicate whether they actually saw the target or whether they guessed the answer, yielding a measurement of awareness. All responses were made with the index and middle fingers of the right hand (index finger for the square and middle finger for the diamond for the first question and index finger for aware and middle finger for unaware for the second one). Each session started with a practice run of 520 trials, and subjects performed 8 blocks of 98 trials for a total of 784 trials.

Since the objective of the current study was to assess differences in awareness when performance was kept constant for physically identical stimuli, we compared correctly identified stimuli which differed in awareness. We therefore first identified the ISI at which subjects had roughly equal numbers of aware and unaware correct trials. The paradigm was validated in a behavioral pretest in which we tested 7 ISIs (13, 26, 39, 52, 65, 78 and 104 ms) with 8 subjects. This behavioral experiment showed that most subjects had equal numbers of aware and unaware correct trials at ISIs 39, 52 and 65 ms. We used those ISIs in the subsequent EEG experiment in addition to an easily visible condition (104 ms) in order to reduce frustration.

### EEG recording and raw data processing

The EEG was continuously recorded from 256 carbon-fiber coated Ag/AgCl electrodes using a Hydrocel Geodesic Sensor Net®. The EEG was digitized at 1 kHz with a band-pass filter of 0–100 Hz and a recording reference at the vertex; impedances were kept below 30 kΩ. Electrodes located on the cheeks and in the nape were excluded and 204 electrodes were maintained for subsequent analysis. Before selecting the relevant epochs, the EEG was re-referenced to the common average reference and digitally filtered between 1 and 30 Hz. We used a 2nd order Butterworth filter with a −12 db/octave roll-off; the filter was computed linearly with two passes, one forward and one backward in order to eliminate phase shifts and with poles calculated each time to the desired cut-off frequency. We extracted epochs of 50 ms before stimulus onset for CA and Correct Unaware (CU) conditions at each subject’s ideal ISI condition, and trials contaminated by oculomotor and other artifacts were excluded. For each participant, channels exhibiting substantial noise were interpolated using a 3D spherical spline interpolation procedure (Perrin et al., 1989). On average, 6.3 channels were interpolated for each subject. The analysis was performed using the Cartool software by Denis Brunet.^1^

### Analysis of pre-stimulus microstates

As mentioned above, the topography of the scalp electric field remains quasi-stable for brief periods of ∼80–120 ms, the so-called EEG microstates (Lehmann et al., 1987; Koenig et al., 2002). During these periods of stability, only the strength, but not the topography of the field can change. The strength of the scalp field is reflected in the Global Field Power (GFP), which is computed as the spatial standard deviation of the potential field (Lehmann and Skrandies, 1980; Skrandies, 1990). Local maxima of the GFP are hence the best representative of a given microstate in terms of signal-to-noise ratio. Previous studies have shown that only the microstate immediately before stimulus onset is crucial for the determination of the fate of an upcoming stimulus (Kondákor et al., 1995; Kondakor et al., 1997; Lehmann et al., 1998; Mohr et al., 2005; Britz et al., 2009, 2011), which is why we restricted our analysis to the microstate immediately before stimulus onset. The microstate analysis comprised five steps:

First, we determined for each subject the ISI at which there were a similar number of trials in the CA and CU conditions.

Second, we extracted the topographic map at the GFP maximum closest to stimulus onset in the 50 ms time window before stimulus onset. Because the topography remains stable for ∼100 ms with abrupt transitions between subsequent states, we reasoned that the GFP peak closest to stimulus onset in the 50 ms time window before stimulus onset was the best representative of the pre-stimulus microstate in a given trial. We did this for the CA and CU conditions for each subject.

Third, we jointly submitted the pre-stimulus microstate maps from all subjects in the CA and CU conditions to a *k*-means spatial cluster analysis (Pascual-Marqui et al., 1995) to identify the templates of the most dominant microstate maps in the two conditions. We wanted our analysis to be strictly data-driven and made no a priori assumptions regarding the number of clusters or the amount of global explained variance (GEV). We performed a cluster analysis with 20 different solutions ranging from 1 to 20 clusters and determined the best solution by means of the minimum of the cross-validation criterion (CV). The CV is a measure of predictive residual variance, i.e., the difference between the data and the model, and its minimum identifies the solution for which the residual variance is minimal or—in other words—the minimum number of clusters that best explain the data.

Fourth, we computed a strength-independent spatial correlation between the template maps representing the optimal solution of the cluster analysis and the topographic map of the single trials. We matched, i.e., labeled each single trial pre-state microstate map with the template map it best corresponded with, thereby assessing its GEV. The GEV is the sum of the explained variance weighted by the GFP. It is a measure of how well a map explains the data both in terms of strength and in terms of frequency of occurrence. This was done to determine how well the templates identified by the cluster analysis are represented in the raw data of each subject.

Fifth, we finally determined which maps dissociated the CA and the CU conditions by statistically comparing their GEV between these conditions.

### Analysis of pre-stimulus source differences

We extracted the single trials labeled by the templates of the maps that dissociated the CA and CU conditions and estimated the magnitude of their intracranial generators with a local autoregressive average (LAURA) inverse solution (Grave de Peralta Menendez et al., 2004). LAURA was computed with a locally spherical realistic head model (LSMAC; Brunet et al., 2011) using the ICBM 152 non-linear atlas of the Montreal Neurological Institute (MNI; Fonov et al., 2011) as the standard brain for all subjects. The LSMAC model does not require the estimation of a best fitting sphere. Instead, it uses the realistic head shape and estimates the local thickness of scalp, skull and brain underneath each electrode. Then, these thicknesses are used in a 3-shell spherical model with the local radii, which allows taking into consideration the real geometry between the electrodes and the solution points. First, the brain surface was extracted from this atlas, and then the gray matter was extracted from the brain. A total of 4766 solution points was regularly distributed in the gray matter of the cerebral cortex and limbic structures. The forward problem was solved with an analytical solution with a 3-layer conductor model. This somewhat simplified realistic head model allows an accurate and rapid analytical solution of the forward problem. It has been shown to give similar results to boundary element head models (Guggisberg et al., 2011). Numerous experimental and clinical studies have shown that this model provides reliable and accurate estimations of intracranial currents (Brodbeck et al., 2009, 2011; Groening et al., 2009; Vulliemoz et al., 2009; Plomp et al., 2010).

When considering estimations of intracranial current distributions, one is faced with the problem of thresholding. There cannot be a predefined threshold that indicates when an estimated source can be reliably considered as “active”. One way of overcoming this is by statistically comparing the estimated intracranial currents between conditions (James et al., 2008, 2011; Britz et al., 2009, 2011; Plomp et al., 2009, 2010; Britz and Michel, 2010). Comparable to statistical parametric mapping used in fMRI research, we statistically compared the estimated intracranial currents in the CA and CU conditions at every solution point. We did this analysis twice: once using only those trials that were labeled as the microstate maps that dissociated the CA and CU conditions and once using all trials irrespective of their microstate labeling. For all analyses reported in the manuscript, we used the False Discovery Rate (Benjamini and Hochberg, 1995) to control for multiple comparisons.

### Analysis of pre-stimulus power and phase

In addition, we analyzed local pre-stimulus differences in alpha power and phase in order to relate our findings of the microstate analysis to those of previous studies (Ergenoglu et al., 2004; Hanslmayr et al., 2005, 2007; Busch et al., 2009; Mathewson et al., 2009). In order to assess the distribution of phase angles and lags, we performed the analysis at all 204 electrodes, and in order to assess the effect of the chosen reference on the distribution of the phase angles and lags, we repeated the phase analysis using five different references: the average reference, averaged mastoids, FPz, Cz and Oz. We applied a discrete Fourier Transform with a Blackman window to the raw EEG in the 200 ms window before stimulus onset and extracted the power and phase for every trial at all electrodes. With a sampling rate of 1000 Hz and a time window of 200 ms, the frequency resolution of the FFT is 5 Hz. We compared the power at 10 Hz in the pre-stimulus period for all trials and those trials classified as the microstate maps that dissociated the CA and CU conditions. For the analysis of phase, we first computed the mean phase angle for each subject in the CA and CU conditions at each electrode. We then assessed where these phases differed significantly using a Watson-Williams test (Watson and Williams, 1956) as implemented in the CircStat Matlab toolbox (Berens, 2009).

## Results

### Behavioral Results

#### Pilot study

**Figure 2:**
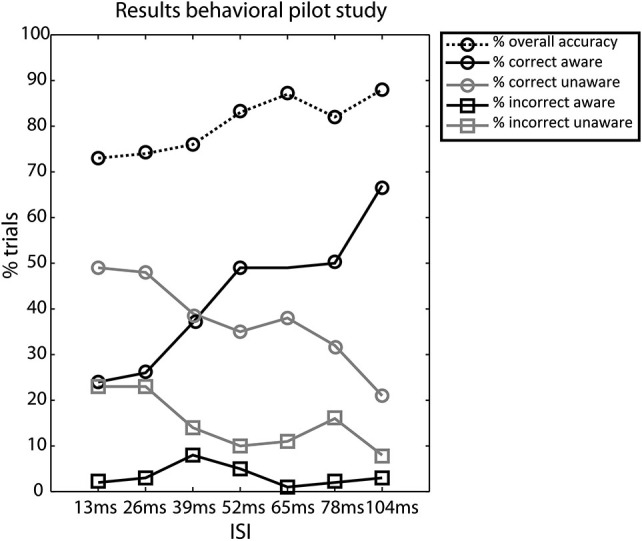
**Results of the behavioral pilot experiment**. Percentage of trials for the different conditions—overall accuracy, correct aware, correct unaware, incorrect aware, and incorrect unaware. Circles denote correct responses and squares denote incorrect responses. Black lines denote aware responses and gray lines denote unaware responses, the dotted line denotes the overall accuracy. Most subjects had equal numbers of correct aware and correct unaware trials in the 39 ms ISI condition.

We assessed awareness as a function of accuracy at the 7 ISIs and the majority of subjects showed equal numbers of trials in the CA and CU condition at an ISI of 39 ms, the results are plotted in Figure 2.

#### EEG study

**Figure 3:**
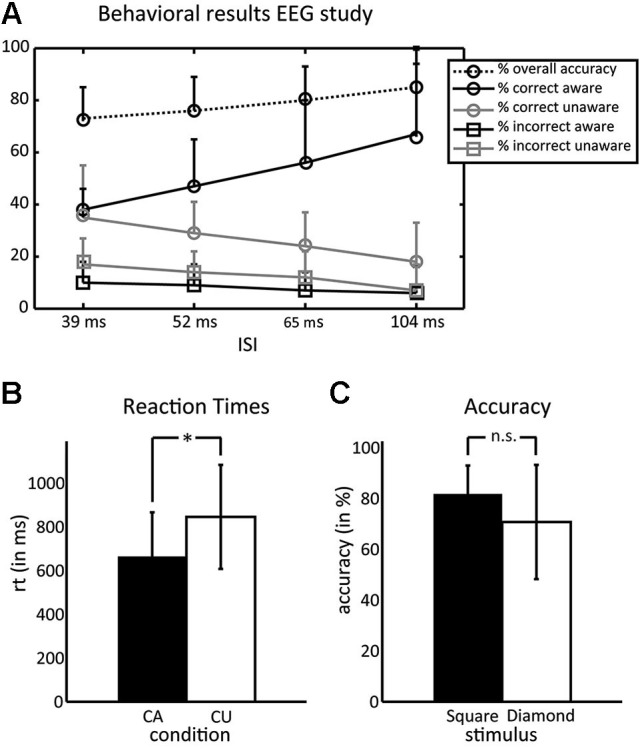
**Behavioral results of the EEG experiment**. **(A)** Percentage of trials for the different conditions—overall accuracy, correct aware, correct unaware, incorrect aware, and incorrect unaware. Circles denote correct responses and squares denote incorrect responses. Black lines denote aware responses and gray lines denote unaware responses, the dotted line denotes the overall accuracy. Most subjects had equal numbers of correct aware and correct unaware trials in the 39 ms ISI condition. **(B)** Reaction times were faster in the CA than the CU condition. **(C)** Subjects did not differ in their ability to correctly identify squares and diamonds.

Figure 3 summarizes the behavioral results. The majority of subjects (75%) showed similar numbers of trials in the CA and CU condition at an ISI of 39 ms, 16.7% of subjects at an ISI of 52 ms and 8.3% at 65 ms. Performance was well above chance at each of these ISIs (73, 76 and 80%, respectively). On average, subjects had 40% (*SD* = 11) of trials in the CA condition and 34% (*SD* = 9) of trials in the CU condition, this difference was not significant (*t*_(1,10)_ = 1.06, *p* = 0.31). Mean reaction times were 665 ms (*SD* = 212 ms) in the CA condition and 819 ms (*SD* = 230) in the CU condition. This difference was significant (*t*_(1,10)_ = 3.8, *p* = 0.0036). Although some subjects reported having seen more squares than diamonds or vice versa, there was no difference in the identification accuracy for both types of stimuli (squares: 81% (*SD* = 11), diamonds: 70% (*SD* = 22); *t*_(1,10)_ = 1.52, *p* = 0.15). There were no learning effects in the main EEG experiment: neither accuracy rates (*F* < 1) nor awareness (*F*_(1,7)_ = 1.33, *p* = 0.249) differed between blocks.

### Pre-stimulus microstates

**Figure 4:**
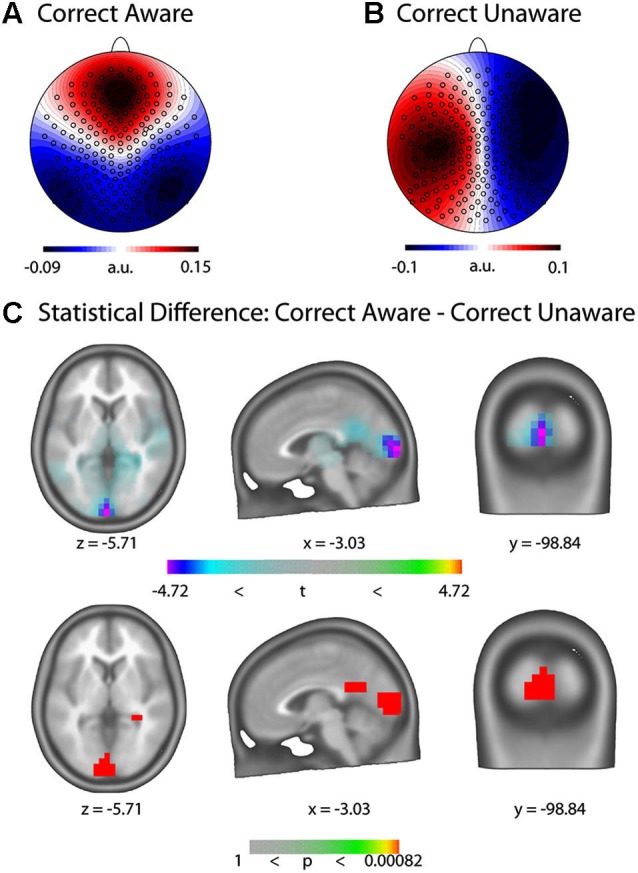
**Pre-stimulus EEG microstate results. (A)** Template of the microstate map for the Correct Aware (CA) condition (Map 16). **(B)** Template of the microstate map for the Correct Unaware (CU) condition (Map 3). The templates represent normalized voltage maps and are hence unit-free. **(C)** Statistical parametric maps (top: *t*-values, bottom: *p*-values) of the LAURA source difference rendered on the ICBM 152 non-linear atlas of the Montreal Neurological Institute (MNI). Blue-violet values indicate increased current density in the CU condition.

After artifact rejection, on average 152 trials were retained for every subject. The GFP peak closest to stimulus onset occurred on average 12.71 ms before the stimulus. The pre-stimulus microstate maps at the GFP peak closest to stimulus onset of these trials were submitted to a *k*-means spatial cluster analysis. The cross validation criterion yielded 16 maps as the best solution which explained 76.26% of the Global Variance. We then computed a strength-independent spatial correlation between the template maps identified in the cluster analysis and those of each trial and statistically assessed which template maps best dissociate the CA and CU conditions. Two microstate maps dissociated the CA and the CU condition with respect to GEV; their templates are displayed in Figures 4A and 4B, respectively. Map 3 had a significantly higher GEV in the CU than the CA condition (*t*_(1,10)_ = −2.67, *p* = 0.0234) and Map 16 had a significantly higher GEV in the CA than the CU condition (*t*_(1,10)_ = 2.98, *p* = 0.014). On average, 15 (+/− 2.8) % of trials were classified as Map 3 or Map16.

### Pre-stimulus source differences

We computed distributed LAURA inverse solutions for trials classified as microstate maps 3 and 16 and assessed their statistical difference at every solution point (Figure 4). We found statistically significant increased activity in bilateral Cuneus and Lingual Gyrus in the CU compared to the CA condition (MNI coordinates of maximal difference: *x* = −3.03, *y* = −98.08,*z* = −5.7, *t* = −4.72, *p* = 0.00082). When considering all trials of the CU and CA conditions irrespective of their microstate map classification, we found no differences in current density anywhere in the brain.

### Pre-stimulus power and phase differences

**Figure 5:**
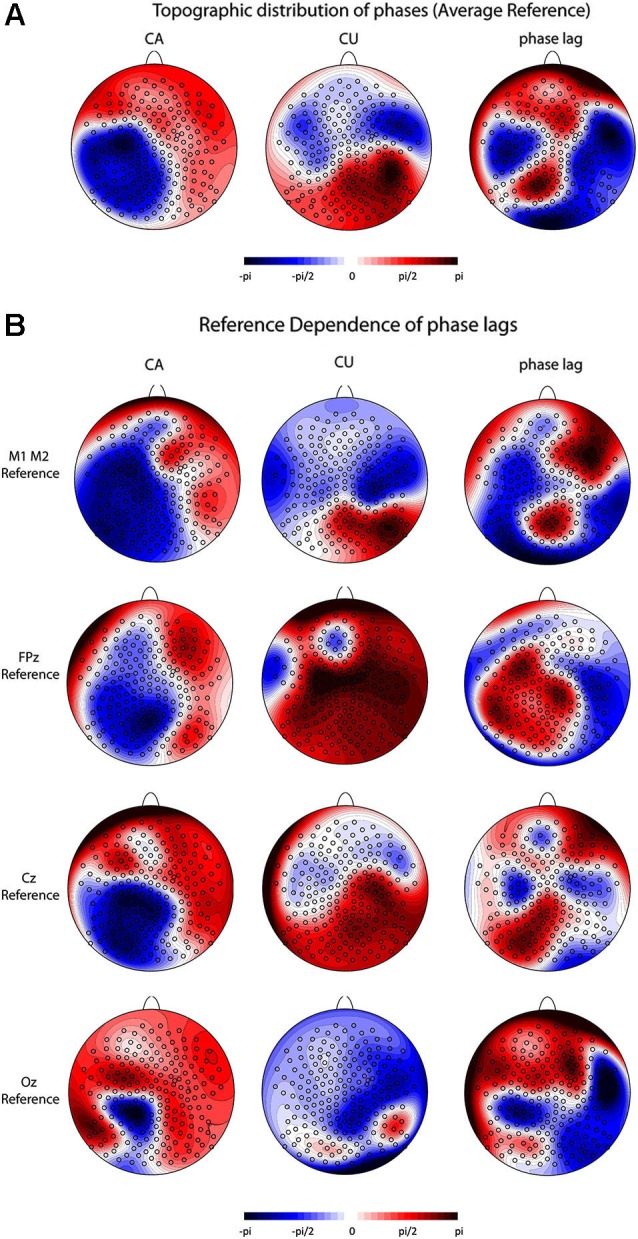
**Pre-stimulus alpha phase results. (A)** Topographic distribution of the phase angles and phase lags for all trials (scaled between –pi and pi) at all 204 electrodes using an average reference for the CA condition (left panel), the CU condition (center panel) and the lag between CA and CU conditions (right panel). **(B)** Reference dependence of the distribution of phase angles and phase lags. The left column depicts the distribution of phase angles in the CA condition, the middle column depicts the distribution of phase angles in the CU condition and the right column depicts the distribution of the phase lags between the CA and the CU conditions. The first row depicts the results for an average mastoid reference, the second row for an FPz reference, the third row for a Cz reference and the fourth row for an Oz reference.

We found no pre-stimulus power differences at 10 Hz. This holds for all trials as well as for trials classified as microstate maps 3 and 16. Figure 5 displays the results of the phase analysis. Panel 5a shows the topographic distribution of the phase angles in the CA and CU conditions (left and middle panels) and the phase lags (the difference of the phase angle in the CA and CU conditions) at all electrodes (right panel). We found significant phase differences between the CA and CU conditions at 94 out of 204 electrodes. Nearly opposite phase angles (phase lags of >170°) were found at only 13 out of 204 electrodes. At five of those electrodes (107, 115, 129, 131, 205), phase lags were >170°, and at the other eight (49, 100, 122, 139, 142, 143, 149, 211), the phase lags were < −170°.

The topographic distribution of the phase angles in the CA and CU conditions and the phase lags as well as the location of significant phase lags depended strongly on the chosen reference (Figure 5c). For an average mastoid reference, we found significant phase differences >170° at five electrodes (149, 152, 157, 193) and significant phase differences < −170° at another five (118, 147, 156, 158, 210) occipital electrodes. For an FPz and a Cz reference, there were no significant phase differences, and with an Oz reference, we found significant differences >170° at 18 electrodes (1, 50, 51, 53, 56, 57, 68, 70, 106, 116, 123, 124, 142, 156, 163, 205, 214, 223) and significant differences < −170° at 12 electrodes (74, 85, 105, 113, 173, 181, 204, 210, 211, 212, 221, 222) at frontal, central and occipital sites.

## Discussion

We show that differences in visual awareness of physically identical stimuli can be related to differences in pre-stimulus microstates and their concomitant neuronal generators. We used a metacontrast masking paradigm in which subjects had to discriminate between a square and a diamond target followed by a mask and compared physically identical stimuli that were correctly identified with and without awareness. We identified two global brain states indexed by the pre-stimulus microstate on a trial-by-trial basis that dissociated the CA and the CU conditions. Statistical parametric mapping of their concomitant intracranial generators revealed increased current density in the Cuneus and Lingual Gyrus before the onset of stimuli that were identified without awareness. These anatomically defined areas are part of the primary visual cortex. Because different topographies necessarily imply different generators (Helmholtz, 1853; Vaughan, 1982), these results indicate that primary visual cortex is more strongly pre-activated when subjects fail to become aware of a stimulus presented at the threshold of awareness.

This finding might initially sound counterintuitive, since one might assume that “more activity” directly implies “better performance” or “increased awareness”, which is likely the case for above threshold stimuli but not necessarily for near-threshold stimuli. Our interpretation of this finding is that the pre-activation of visual cortex apparently interferes with adequate processing of weak stimuli. The effects of masking are commonly explained by disruption of re-entrant processing between higher and lower visual areas (Fahrenfort et al., 2007, 2008) and recurrent processing within early visual areas (Boehler et al., 2008) by the mask after the stimulus is encountered. We hypothesized that the pre-stimulus brain-state can also influence the efficiency of masking and could identify two pre-stimulus brain states indexing differential activity in early visual cortex that dissociate efficient from inefficient masking. Our results can complement the prevailing view of the mechanisms underlying masking: the pre-stimulus activity in early visual cortex can be considered as an alternative source of interference with re-entrant processing. An alternative explanation is that if the primary visual cortex is already active before the onset of a weak stimulus at the threshold of awareness, such a weak stimulus cannot provide sufficient additional activity to attain awareness. In other words, the brain appears to be unable to distinguish between the spontaneous pre-activation of primary visual cortex and the post-stimulus activity evoked by a weak near-threshold stimulus. This finding is supported by a recent study by He (2013) where she elegantly shows how behaviorally relevant negative interactions between pre- and post-stimulus activity can be observed on a trial-by-trial basis in the absence of mere amplitude differences between conditions. This inverse relation between pre- and post-stimulus activity sheds a new light on the functional relation between spontaneous and evoked activity. In another modality, trial-to-trial differences in output force have been found to be inversely related to levels of pre-stimulus activity in primary motor cortex (Fox et al., 2007). These results underline the importance of recent methodological advances that consider trial-to-trial variations in ongoing activity instead of averaged differences between conditions which reveal important new insights into brain function. Traditionally, trial-to-trial variations in behavioral and neuronal measures are dismissed as noise and eliminated by signal averaging, however, it is becoming increasingly evident that these variations are functionally significant activity with an important impact on perception and cognition (Mohr et al., 2005; Fox et al., 2007; Britz et al., 2009, 2011; Britz and Michel, 2010, 2011; Garrett et al., 2011, 2013; Kanai and Rees, 2011; Tzovara et al., 2012, 2013; He, 2013). Our current results further support the significance of such apparently slight trial-to-trial variations; even though we considered only a subset of trials, we identified that proportion which yielded consistent differences across all single trials from all subjects. We of course can not rule out that activity differences in other brain areas—most probably in parietal and frontal areas—might have also contributed to the differences in the emergence of perceptual awareness. However, the contributions of other brain areas are less strong and less consistent than those in early visual cortex immediately before stimulus onset.

In order to compare our results to previous studies, we assessed pre-stimulus alpha power and phase which are considered to index different aspects of cortical excitability. Alpha power is considered as an index of alertness and general excitability of visual cortex to which it is inversely related (Pfurtscheller, 1992). Alpha phase on the other hand is assumed to reflect cyclic variations in cortical excitability (Busch et al., 2009; Mathewson et al., 2009; Scheeringa et al., 2011). Several EEG and MEG studies have shown increased pre-stimulus alpha power for undetected compared to detected stimuli (Ergenoglu et al., 2004; Hanslmayr et al., 2007; Romei et al., 2008a, b, 2010). Likewise, the ability to correctly distinguish between two stimuli presented at the discrimination threshold depends on the pre-stimulus alpha power (Hanslmayr et al., 2005; van Dijk et al., 2008). Simultaneous EEG-fMRI studies however provide mixed results about the relation between alpha power and activity in primary visual cortex indexed by the BOLD response (Becker et al., 2011; Scheeringa et al., 2011). In the present study, we found no pre-stimulus differences in alpha power at 10 Hz. This is surprising given that other studies have shown that both awareness and discrimination ability can vary as a function of pre-stimulus alpha power. In those studies, performance was close to chance, i.e., subjects were able to detect or to correctly discriminate stimuli in roughly 50% of cases and in such cases alpha power appears to be a powerful tool to distinguish between differences in detection or discrimination ability. Here, we analyzed differences in awareness for correctly identified stimuli, and performance was very high (subjects responded correctly in about 80% of trials). When performance is close to ceiling, alpha power no longer appears to be a good parameter to distinguish between correct discrimination with and without awareness. Instead, we could relate the differences in awareness for correct target discrimination to a global pre-stimulus brain state that reflects differential pre-stimulus activity in primary visual cortex.

Other studies have related awareness to local differences in phase of the alpha and theta band (Busch et al., 2009; Mathewson et al., 2009; Dugué et al., 2011). These differences in awareness as a function of the pre-stimulus alpha phase, i.e., that a stimulus was perceived when it occurs during a certain phase and that it was not perceived during the opposite phase, were interpreted as cyclic variations of cortical excitability or inhibition. However, this claim is difficult to support because local variations in phase are reference dependent, which renders the functional interpretation of a peak or trough very challenging. We analyzed the pre-stimulus alpha phase at all 204 electrodes using five different references. We found significant phase inversions between the CA and CU conditions, but both their location and their direction varied strongly with the chosen reference. Not a single electrode out of the 204 showed consistent phase inversions across the five references we used, which renders the functional interpretation of the location of phase differences on the one hand and that of peaks and troughs at best arbitrary. We thus replicate the results from previous studies that show differences in awareness as a function of pre-stimulus alpha phase, but we also show that such local phase differences have to be interpreted with a lot of caution.

The link between visual cortex excitability and alpha phase has been claimed without a direct demonstration; differences in excitability are generally inferred from the fact that a stimulus is perceived or not. Here, we show that this link between local phase, awareness and pre-stimulus activity in primary visual cortex is not as direct as previously claimed. We show that the global brain state immediately before stimulus onset can be more unambiguously linked to pre-stimulus differences in primary visual cortex activity than local differences in alpha phase, and the present results corroborate the importance of the state of visual cortex at the time of stimulus arrival for visual awareness.

The present results extend the results from our prior studies in which we showed that the *changes* in the perceptual awareness for ambiguous stimuli and during binocular rivalry arise as a direct consequence of pre-stimulus microstates (Britz et al., 2009; Britz and Michel, 2011). These studies revealed that the right inferior parietal cortex is implicated in the generation of perceptual reversals of multi-stable stimuli and that inferior temporal areas are involved in percept stabilization during binocular rivalry. Here, we show that the *emergence* of perceptual awareness for correctly identified stimuli presented at the threshold of awareness can likewise be linked to the pre-stimulus microstate which indexes that primary visual cortex is differentially active immediately before stimulus onset.

Activity in primary visual cortex is necessary but not sufficient to attain awareness (Tong, 2003), and there is ample evidence that the dynamic interplay of activity in lower visual and higher order brain areas in parietal and frontal cortex are crucial for awareness (Lumer et al., 1998; Dehaene et al., 2006; Lamme, 2006; Lau and Passingham, 2006). Using fMRI, Lau and Passingham (2006) have shown that prefrontal cortex comes into play when subjects become aware of stimuli that are equated for performance but not physical identity, thus confounding awareness and stimulus properties. Because of the slow temporal dynamics of the hemodynamic response function, the precise temporal allocation of fMRI effects remains a challenge. Several EEG studies however indicate that parietal and prefrontal areas might come into play only after stimulus onset (Sergent et al., 2005; Fahrenfort et al., 2007, 2008; Genetti et al., 2010) when subjects become aware of stimuli.

In the present study, we bridged the gaps between awareness, accuracy and physical identity by assessing awareness when accuracy was kept constant for physically identical stimuli. For every subject, we compared physically identical stimuli that were correctly identified but that differed in awareness. To our knowledge, this is the first study that has attempted to equate both physical identity and behavioral accuracy when assessing differences in awareness. The apparent differences between awareness and accuracy during experimental manipulations of stimulus visibility have been recently challenged as being due to conservative response criteria for the awareness ratings and by being abolished by using the bias-free measurement of d’ (Ko and Lau, 2012; Lloyd et al., 2013). It should be noted though, that the inclusion of non-stimulus trials necessary for the computation of d’ themselves might introduce more conservative response criteria because subjects have to distinguish between stimuli with different degrees of visibility and the physical absence of stimuli. Furthermore, the order of identity and awareness judgments might likewise influence the awareness ratings. In the present study, subjects knew that there was always a stimulus present and that they had to indicate whether or not they saw it after they indicated its identity which should not have strongly biased their awareness judgment. However, future studies are needed to address these issues in more detail.

Taken together, the same physical stimuli can undergo very different perceptual fates as a function of the state of the brain before stimulus arrival: differences in frequency power or phase on the one hand, and differences in the overall configuration of intracranial generators indexed by the scalp topography on the other hand yield different perceptual outcomes of the same stimulus. These findings are important to consider when comparing ERPs to differences in perceptual awareness: differences in topography, power or phase between single trials in the “baseline” period can be easily eliminated and translated into a post-stimulus effect by performing a baseline correction.

Previous studies have claimed that differences in awareness result from differences in pre-stimulus alpha power or opposite pre-stimulus alpha phase, which supposedly reflect cyclic variations in the excitability of primary visual cortex. However, a direct demonstration between alpha power, alpha phase, visual awareness, and activity in primary visual cortex has been lacking. In the present study, we show that differences in awareness for the same stimuli arise from differences in a global pre-stimulus brain state that reflects differential pre-stimulus activity in primary visual cortex.

## Author Contributions

Juliane Britz, Laura Díaz Hernàndez and Tony Ro, designed research. Juliane Britz and Laura Díaz Hernàndez performed research. Juliane Britz and Laura Díaz Hernàndez analyzed the data, Juliane Britz, Laura Díaz Hernàndez, Tony Ro, and Christoph M. Michel wrote the manuscript.

## Conflict of interest statement

The authors declare that the research was conducted in the absence of any commercial or financial relationships that could be construed as a potential conflict of interest.

## Footnotes

^1^https://sites.google.com/site/fbmlab/cartool
